# Quantitatively Measuring Privacy in Interactive Query Settings Within RDBMS Framework

**DOI:** 10.3389/fdata.2020.00011

**Published:** 2020-05-05

**Authors:** Muhammad Imran Khan, Simon N. Foley, Barry O'Sullivan

**Affiliations:** ^1^Department of Computer Science, Insight Centre for Data Analytics, University College Cork, Cork, Ireland; ^2^Department of Information Security and Communication Technology, Norwegian University of Science and Technology, Gjøvik, Norway

**Keywords:** electronic privacy, data analtyics, relational database management systems (RDBMS), privacy score, n-grams

## Abstract

Little attention has been paid to the measurement of risk to privacy in Database Management Systems, despite their prevalence as a modality of data access. This paper proposes *PriDe*, a quantitative privacy metric that provides a measure (privacy score) of privacy risk when executing queries in relational database management systems. PriDe measures the degree to which attribute values, retrieved by a principal (user) engaging in an interactive query session, represent a reduction of privacy with respect to the attribute values previously retrieved by the principal. It can be deployed in interactive query settings where the user sends SQL queries to the database and gets results at run-time and provides privacy-conscious organizations with a way to monitor the usage of the application data made available to third parties in terms of privacy. The proposed approach, without loss of generality, is applicable to BigSQL-style technologies. Additionally, the paper proposes a *privacy equivalence relation* that facilitates the computation of the privacy score.

## 1. Introduction

The recent past has witnessed an exponential increase in the amount of data being collected by contemporary organizations. Data analytics offers a broad spectrum of benefits. It enables contemporary organizations to anticipate business opportunities and to deliver relevant products to their customers, gives them a competitive advantage, facilitates cost reduction, allows personalization of service, and results in improvements in the customer experience. A long-term impact of data analytics is, likewise, that it enables early detections of outbreaks of diseases; in a nutshell, analytics over a huge amount of data is likely to result in an incredible impact on businesses and our society. Data comes from multiple sources and comprises personal and sensitive data. On the one hand, one cannot deny the importance and value of data, but, on the other hand, the fact that data can include personal and sensitive items means that the usage and storage of and access to this data raise security and privacy concerns. Contemporary organizations grant access to their data to third parties who specialize in data analytics so as to gain richer insight from their data. These organizations are also increasingly conscious of the privacy of the data of individuals. Therefore, privacy controls that monitor the usage of data in the privacy sense are exceedingly desirable for contemporary organizations.

In this paper, we propose a privacy control based on the run-time measurement of privacy risk. The proposed metric, in essence, is a Privacy Distance (PriDe) between the past and the current querying behavior of an analyst. Systematically measuring and quantifying privacy risk is non-trivial (Becker and Chen, 2009; Wagner and Boiten, 2017). The focus of the majority of the metrics in the literature has been on social-networking environments or on “open” systems where the actions of the users or consumers of the data tend not to be constrained. However, less attention has been paid to approaches that objectively measure privacy risk within the Relational Database Management System (RDBMS) framework. The contribution of this paper is the construction of a metric that objectively measures privacy risks by providing data curators with a score within the RDBMS framework. We are interested in providing a real-time measure of the (privacy) risk associated with queries by third parties (Users or analysts^1^) against a Relational Database. PriDe computes a run-time score of the privacy risk that arises from a query to the database. It is a measure of the degree to which attribute values, retrieved by a principal engaged in an interactive query session, may represent a reduction of privacy with respect to the attribute values previously retrieved by the principal from the RDBMS. Intuitively, the score provides the basis for a form of privacy anomaly detection: to what degree of privacy do the current queries of a user differ from the “normal” past queries of the user. The proposed approach uses n-gram profiles to model the querying behavior of the principals (users). N-grams have been effectively used to identify short-term correlations between events in logs, thus enabling us to build an approximation of the system behavior (Forrest et al., 1996). Thus, n-grams have the potential to capture patterns reflecting the attempted inferences made for some attribute values. Once n-gram profiles are generated, a comparison of the profiles can be carried out for the score computation. Moreover, a case of cold start is also studied, where a profile to compare against (baseline profile) is not available. Additionally, to enable the comparison to be carried out in terms of privacy, a *privacy equivalence relation* is also proposed in this paper. This paper is a significant revision of the paper (Khan et al., 2019c) and extends this work, with the treatment of cold-start (absence of baseline profile - past querying behavior), experimental results pertaining to the evaluation of the cold-start scenario and includes discussions, for instance, on the a use-case of computing privacy score.

The remainder of this paper is organized as follows. We present related work in section 2. Section 3 describes the design of PriDe, the privacy score model. Cumulative privacy score is presented in section 3.3, followed by the demonstration of the model in section 4. Some conclusions have been drawn in section 5.

## 2. Related Work

A large portion of the literature in the context of privacy research focuses on the anonymization of the data in the databases. We have witnessed several definitions of privacy including *k*-anonymity (Sweeney, 2002), *l*-diversity (Machanavajjhala et al., 2007), *t*-closeness (Li et al., 2007), and differential privacy (Dwork, 2008). Syntactic definitions of privacy (Clifton and Tassa, 2013), like *k*-anonymity, *l*-diversity, and *t*-closeness, deal with Privacy-Preserving Data Publishing (PPDP), where one can use syntactic privacy definitions to anonymize the data to preserve the privacy of the individuals and subsequently publish the anonymized data. Differential privacy was initially designed for statistical databases. Statistical databases allow aggregated queries and have applications in multiple domains (Deng and Lv, 2015). Differential privacy mechanisms are for interactive settings where the user sends a query and gets a result at run-time. Syntactic definitions and differential privacy aim to anonymize the data rather than providing a quantitative score as an indication of changes in the level of privacy.

Another line of research work is in the context of recommender systems (Shapira et al., 2004; Halkidi and Koutsopoulos, 2011; Arnau et al., 2014). For instance, the work presented in Arnau et al. (2014) ensures the privacy of a user's web searches from surveillance and data-profiling. A profile of a user's interests is represented as a histogram. This user's profile of interests is then compared with a consolidated profile of the interests of the population. Deviation of the user profile from the population's profile is measured using entropy and KL-divergence (both acting as privacy metrics). The approach in Shapira et al. (2004) is aimed at providing a noise-added version of an individual's navigation tracks to prevent an attacker from inferring the individual's profile of interests. The approach generates redundant searches for a variety of fields of interest; in other words, noise in the form of these redundant searches was added in order to confuse the attacker. Profiles in the context of recommender systems are the profiles of interest of individuals and are constructed by considering the individual's search history; for example, where a person frequently searches for sports cars, the personalized information system deduces that the person is interested in sports cars. The keyword “sports cars” then becomes a part of the individual's profile of interests. The notion of the profile of interest is different from the behavioral profile (n-gram profile) presented in our proposed work. The behavioral profile (n-gram profile) captures the querying behavior of an individual while attempting to capture correlations between the queries. An interesting line of research is to measure the degree of anonymity in Anonymous-Communication Systems (ACS), where several approaches have used information-theoretic quantities to evaluate ACSs (Díaz et al., 2003; Serjantov and Danezis, 2003; Diaz, 2006). For instance, the approach in Díaz et al. (2003) and Diaz (2006) measures the degree of anonymity as normalized entropy. Another facet of privacy is manifested in its perseverance while the private data is outsourced, for which promising approaches have been proposed in Pei et al. (2007) and Genga and Zannone (2018).

However, little research has been reported on objectively measuring privacy risks within the Relational Database Management System (RDBMS) framework in settings where the goal is to measure the changes in the level of privacy of the individuals in the database when a user or an analyst accesses it. The primary aim of the past privacy research in the context of PPDP, recommender systems, and ACSs is to anonymize the original data to obtain a distorted version of the original data. The original data is distorted in such a way that the attacker perceiving the distorted version is unable to infer any of the individual's identification information from it. Additionally, the privacy metrics in the context of PPDP, recommender systems, and ACSs are crafted in accordance with the technicalities prevalent in that particular domain. This aim differs from the aim of the proposed work, which is to objectively measure the reduction of privacy of the individuals while the analyst interacts with the database. We are interested in measuring the change in privacy level (reduction in the privacy level) rather than attempting to anonymize the database. Moreover, a novel set of challenges is manifested when one works within the framework of RDBMS. One such example is cases where one SQL query is a subset of another SQL query in terms of privacy.

### 2.1. Measuring Privacy

**Figure 1:**
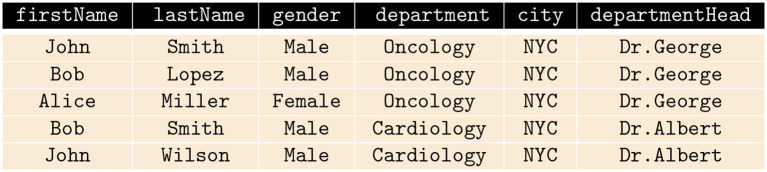
An example table with records of five individuals.

PriDe measures the degree to which attribute values, retrieved by a principal (user) engaging in an interactive query session, represent a reduction of privacy with respect to the attribute values previously retrieved by the principal (user) from the RDBMS. In this work, a user(s) or analyst(s) who has been granted access to the database, potentially has malicious intent. The database is maintained by a contemporary organization (data curator), and the database consists of distinct records of numerous individuals. In order to demonstrate the difference between a naïve calculation of the amount of data released in response to the SQL queries made by an analyst and PriDe, consider a database containing a table with attributes including firstName, lastName, department, gender, city, and departmentHead, as shown in Figure 1. Suppose that the very first five queries that an analyst makes to the table select only the value for the attribute city. A naïve calculation of the amount of data retrieved by the analyst would be five values (the number of records selected) for the attribute city; however, the returned values (NYC) do not affect the privacy of any individual in the database. PriDe calculates the amount of information, through attribute values, released to the analyst in terms of privacy. By “in terms of privacy,” we mean whether or not if this release affects the privacy of any individual. Let's say an analyst makes queries to get values for the attribute gender followed by queries to get values for the attribute department by specifying the condition in the WHERE clause of the SQL statement as WHERE city = “NYC.” We would expect that this behavior would result in an increasing privacy score. Again, if the analyst makes another query asking for the value for the attribute firstName, this further increases the privacy score. However, in the case of PriDe, when, after making these queries, the analyst makes a query asking for the value of attribute city, then this does not increase the privacy score, as this query does not affect the privacy of any individual in the database. Another aspect of computing privacy score is the consideration of “safe” past behavior or “safe” queries. For instance, we know that it is a common occurrence that a query to get the value of the attribute department will be followed by a query to get the value of the attribute departmentHead and vice versa. This sequence of queries, if it appeared at run-time, would not increase the privacy score either while PriDe is deployed.

## 3. PriDe—The Privacy Score Model

In this section, the architecture of PriDe—a privacy score model—is outlined. Intuitively, PriDe can be considered to be a posterior privacy control. The computation of the privacy score is based on behavioral profiles. Behavioral profiles approximate the query behavior of a user. Behavioral profiles are inferred from a DBMS log of the SQL queries (audit logs). The fundamental idea is to capture the normal querying behavior of a user in one profile (*baseline profile*) and capture the posterior querying behavior of the user in another profile (*run-time profile*). A comparison (in a privacy sense) of the *baseline profile* and the *run-time profile* then results in the privacy score. The baseline profile is a profile that is constructed using those queries collected in the audit log for which there are no presumed privacy risks. When we say that there are no presumed privacy risks, we mean that we are making the assumption that the past normal behavior is considered to be risk-free from a privacy perspective; this is the same kind of assumption that is made for conventional anomaly detection systems (Chandola et al., 2009). The baseline profile is constructed before the privacy score system is operational. The run-time profile is a profile constructed after the analyst has been granted access to the information system. In this work, we consider an adversary model where the user or the analyst presumably has malicious intentions. The run-time profile is generated from the audit log consisting of queries made by an analyst. PriDe operates in two phases: 1—the profile construction phase, and 2—the profile comparison phase, as described in the next sections.

### 3.1. Modeling Querying Behavior

The profile construction phase consists of the query abstraction step followed by the construction of an n-gram profile. We discuss both these steps in this section.

#### 3.1.1. SQL Query Abstraction

**Figure 2:**
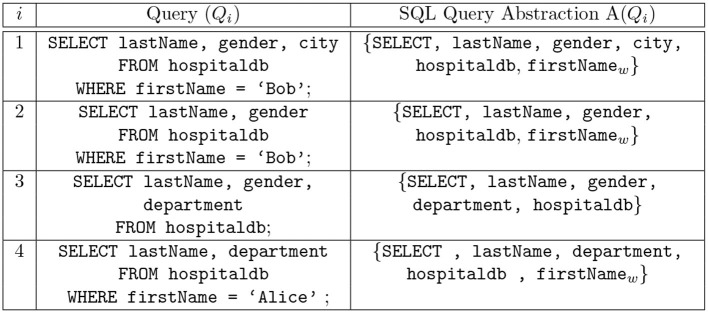
SQL query abstraction: The first element of the query abstraction is the command type. The second element of the query abstraction is attribute names followed by the relation names and the names of the attributes in WHERE clause. To differentiate the attributes queried and the attributes in the WHERE clause, the attributes of the WHERE clause are affixed with a subscript value indicating that the attribute is a part of the WHERE clause.

It is necessary to consider the audit logs of SQL queries at some level of abstraction, for example, in their use for anomaly-based intrusion detection systems to detect insider threats to an organization's DBMS (Lee et al., 2002; Low et al., 2002; Hussain et al., 2015; Kul et al., 2016; Sallam et al., 2016), where an insider is an employee of an organization with legitimate access privileges (Uno et al., 2004; Koh and Rountree, 2005). The use of abstraction, in practice, is considered since audit logs typically encompass a large number of queries. It has been reported in a recent study that within the time period of only 19 h, around 17 million SQL queries were made in a major US bank (Kul et al., 2016). Therefore, a way to summarize the audit logs is desirable. Abstraction is a tuple representation of an SQL query and consists of query features like relation name, attribute names, the amount of returned data, and any statistics on the returned data. One can categorize query features into syntax-centric, data (result)-centric and context-centric features (Mathew et al., 2010; Sallam et al., 2016). For our work, we originally considered a naïve abstraction where only the SQL command type (i.e., INSERT, SELECT, UPDATE) is selected to represent the original SQL query. However, this is too coarse-grained and does not take into account the rest of the queried attributes. In contrast to coarse-grained query abstraction, intuitively, one wants to use a more fine-grained representation of the SQL query by using the entire SQL query to generate a profile. The purpose of query abstraction is lost in the case where a very fine-grained representation (the entire SQL query) is adopted. The idea behind query abstraction is to summarize a log in a meaningful and useful way. In this paper, we use an SQL query abstraction method similar to the one that has already been studied in the context of mining the logs of SQL queries in Makiyama et al. (2015). Some examples of the SQL query abstractions deployed in the paper are depicted in Figure 2. The attribute and relation names are part of the abstraction, along with the SQL command type. The motivation behind using this particularization of SQL query abstraction stems from the privacy-preserving processing. Along with achieving privacy-preserving processing of audit logs, implicitly, we also attain the objective of effectively summarizing the audit logs, so as to have the potential to grow large in size. In any contemporary organization, the levels of access privileges differ for different employees (insiders). For example, a data scientist is authorized to access customer data and on the other hand, a security officer might not be authorized to access the same data. Usually, any other security system is operated under the supervision of roles like privacy or security officers, enabling them to inspect the many instances of operations during the execution of the system. Therefore, it might not be desirable to expose entire queries for inspection. This is undesirable in sensitive environments; nevertheless, in the case of relaxed environments, the entire query abstraction or a more relaxed query abstraction can be used. Privacy-preserving processing of data has many interesting manifestations (Zhang and Zhao, 2007; CERT, 2014). In our work, experiments were performed with other query abstractions (including the entire query). However, discussion on the experiments pertaining to these other query abstractions is not included in this paper due to space limitations. We observed that the query abstractions shown in Figure 2 appear to fit well in our model in contrast with other query abstractions that were considered.

**Definition 3.1. Query Abstraction:** Given a set of SQL queries *Q*, the abstraction *A*(*Q*) of the queries in *Q* is a mapping of *Q* to *A*(*Q*), where the mapping function *A*():*Q* → *A*(*Q*) defines a many-to-one relation.

An abstraction of an SQL query *Q*_*i*_ is denoted as *A*(*Q*_*i*_). The first element of *A*(*Q*_*i*_) provides the command type, that is, SELECT, UPDATE, DELETE, INSERT. The second element of *A*(*Q*_*i*_) is the attribute and relation names of the command. Furthermore, to differentiate the attribute values queried and the attributes in a WHERE clause, the attributes of the WHERE clause are affixed with a subscript “_*w*_” indicating that the attribute occurs in WHERE clause, for example, gender_*w*_.

#### 3.1.2. Constructing Profiles

While n-grams have their origins in computational linguistics (Damashek, 1995; Kalchbrenner et al., 2014) and natural language processing (Sidorov et al., 2014), they are well suited for modeling short-range correlations between events in logs (Forrest et al., 1996). N-gram based models have been frequently used in the literature in the context of intrusion detection systems, where they are found to be effective in capturing normative behavior (Hofmeyr et al., 1998; Somayaji and Forrest, 2000). N-grams are sub-sequences of events generated by sliding a window of size “*n*” over a log of events. When *n* = 2, the resulting sub-sequences/n-grams are known as bi-grams, while in case of *n* = 3 the sub-sequences are known as tri-grams. N-grams are sub-sequences of a given sequence of elements, generated by sliding a window of size “*n”* across the sequence. An n-gram model allows approximation of the query behavior of a user and represents it in the form of a profile of behavior. We refer to behavioral profiles as n-gram profiles. N-gram profiles are inferred from an RDBMS log of the SQL queries (audit logs). Audit logs of application systems have been frequently used in the literature as a basis for mining behaviors (Alizadeh et al., 2018). The n-gram profiles are generated using the approach in (Khan and Foley, 2016; Khan et al., 2019a). To generate an n-gram profile, it is assumed that an RDBMS audit log *L* is available. The audit log *L* is a sequence *Q*_1_, *Q*_2_, *Q*_3_, …, *Q*_*m*_ of SQL queries including SELECT, UPDATE, INSERT, and DELETE statements. The queries in the audit log *L* are a mix of simple queries as well as complex queries that involve joins, GROUP BY statements, HAVING clauses, nested queries, and so forth.

**Definition 3.2. n-gram profile:** Given a sequence *L* of SQL queries (abstractions), the n-gram profile β = *ngram*(*A*(*L*), *n*) is the set of all sub-sequences of size “*n*” that appear in *A*(*L*).

For example, the bi-gram model for the log abstraction, that is, the sequence of query abstractions (*A*(*Q*_1_), *A*(*Q*_1_), *A*(*Q*_4_)) is {〈*A*(*Q*_1_), *A*(*Q*_1_)〉, 〈*A*(*Q*_1_), *A*(*Q*_4_)〉}.

### 3.2. Comparing Profiles

The privacy score, in essence, is a privacy distance that indicates the objective changes between the past and the current querying behavior of the analysts. The proposed approach for computing privacy score enables measurement of the change in privacy by comparing an analyst's past and current querying behavior, with both of the behaviors represented by n-gram profiles. We denote an n-gram profile as β. SQL logs collected for the construction of the baseline n-gram profile and the run-time n-gram profile are denoted as $L_{N}$ and $L_{R}$, respectively. The baseline profile and the run-time profile constructed from these logs are denoted as $\beta_{N}$ = *ngram*(*A*($L_{N}$), *n*) and $\beta_{R}$ = *ngram*(*A*($L_{R}$), *n*), respectively.

**Definition 3.3. Mismatches:** Given a baseline profile and a run-time profile that are compared with each other, the set of mismatches is given by $S_{miss}^{\beta_{N},\beta_{R}}$=$\beta_{R}$ − $\beta_{N}$. Let $\left|S_{miss}^{\beta_{N},\beta_{R}}\right|$ be the number of mismatches or the number of elements in the set $S_{miss}^{\beta_{N},\beta_{R}}$. We denote a mismatched n-gram as $G_{i}^{miss}$ ∈ $S_{miss}^{\beta_{N},\beta_{R}}$.

We need to go beyond the simple comparison of n-gram profiles (subtracting the baseline profile from the run-time profile) and would like to have a more fine-grained comparison at n-gram level and subsequently at the query (attribute) level, the reason being that we are interested in determining the closest match in baseline profile $\beta_{N}$ for the mismatched n-gram $G_{i}^{miss}$. This is because, when we perform a simple subtraction comparison, we tend to make a binary comparison; that is, either the n-gram is the same as other n-gram, or it is not. For instance, consider the following three n-grams *G*_1_ = 〈(SELECT, firstName), (SELECT, department)〉, $G_{2}^{miss}$ = 〈(SELECT, firstName, lastName), (SELECT, department)〉, $G_{3}^{miss}$ = 〈(SELECT, city), (SELECT, gender)〉 where *G*_1_ ∈ $\beta_{N}$ and $G_{2}^{miss}$, $G_{3}^{miss}\in$ $S_{miss}^{\beta_{N},\beta_{R}}$. If we compare $G_{2}^{miss}$ and $G_{3}^{miss}$ against *G*_1_, intuitively, $G_{2}^{miss}$ has some degree of similarity with *G*_1_, while on the other hand, $G_{3}^{miss}$ is entirely different from *G*_1_. Thus, we need to take into account the degree of the similarity of the mismatched n-gram to its closest match if we desire to make a richer comparison between two profiles.

#### 3.2.1. Distance Between N-Grams

In order to find the closest match of the mismatched n-gram $G_{i}^{miss}\in S_{miss}^{\beta_{N},\beta_{R}}$, that is to say, how far $G_{i}^{miss}$ is from its closest match in $\beta_{N}$, a measure to compare two n-grams is desired. To find the closest match, we deploy the strategy of comparing the corresponding SQL query abstraction of two n-grams. In SQL query (abstraction) similarity research, the Jaccard distance (Phillips, 2015) has been commonly used to find similarity between two SQL query abstractions (Stefanidis et al., 2009; Aligon et al., 2014; Kul et al., 2018). The Jaccard distance is expressed as follows:

(1) $$\begin{array}{l} JaccardD\left(X,Y\right)=\left(\left|X\cup Y|-|X\cap Y\right|\right)/|X\cup Y| \end{array}$$

where *X* and *Y* are SQL query abstractions. In order to compare two query abstractions using the Jaccard distance, we have to consider an SQL abstraction as a set for comparison.

**Definition 3.4. Distance Function:** We define the function for the comparison of two n-grams as *Dist*(*G*_*i*_, *G*_*j*_) = $\sum_{r=1}^{n}JaccardD\left(G_{i_{r}},G_{j_{r}}\right)$, where *r* is the index (position) of the item in the n-gram. N-grams of length *n* result in *n* comparisons of SQL query abstractions. The value of *Dist*(*G*_*i*_, *G*_*j*_) falls in the interval [0, *n*], where the value is 0 if two n-grams are identical and the value is *n* if two n-grams of length *n* are distinct.

If two SQL queries abstractions are entirely dissimilar, then the similarity value is 1, and if they are exactly the same, then the similarity value is 0. Consider $S_{miss}^{\beta_{N},\beta_{R}}$ = {$G_{1}^{miss}$, $G_{2}^{miss}$, …, $G_{k}^{miss}$ }, and $\beta_{N}$ = {*G*_1_, *G*_2_, …, *G*_*m*_}. Each n-gram $G_{i}^{miss}\in S_{miss}^{\beta_{N},\beta_{R}}$ is compared with each n-gram *G*_*i*_ in the baseline profile $\beta_{N}$. This results in a total number of *k* × *m* comparisons, that is, $\langle Dist(G_{1}^{miss}$, *G*_1_), $Dist(G_{1}^{miss}$, *G*_2_), $Dist(G_{1}^{miss}$, *G*_3_), …, $Dist(G_{1}^{miss}$, *G*_*m*_)〉, $\langle (G_{2}^{miss}$, *G*_1_), $Dist(G_{2}^{miss}$, *G*_2_), $Dist(G_{2}^{miss}$, *G*_3_), …, $(Dist(G_{2}^{miss}$, *G*_*m*_)〉, …, $\langle Dist(G_{k}^{miss}$, *G*_1_), $Dist(G_{k}^{miss}$, *G*_2_), $Dist(G_{k}^{miss}$, *G*_3_), …, $Dist(G_{k}^{miss}$, *G*_*m*_)〉. We denote each iteration as *Iter*_*l*_ = $\langle Dist(G_{i}^{miss}$, *G*_*j*_), $Dist(G_{i}^{miss}$, *G*_*j*+1_), $Dist(G_{i}^{miss}$, *G*_*j*+2_), …, $Dist(G_{i}^{miss}$, *G*_*j*+*m*_)〉. A single iteration here is defined as the comparison of one n-gram from $S_{miss}^{\beta_{N},\beta_{R}}$ with every n-gram in $\beta_{N}$. We take the minimum value of *Dist* from each iteration, i.e., *Min*(*Iter*_*l*_) that belongs to the interval [0, *n*]. Subsequently, the summation of all *Min*(*iter*_*l*_) values results in a privacy score. Given two n-gram profiles $\beta_{N}$ and $\beta_{R}$, the privacy score is computed between these n-gram profiles as $P_{\langle \beta_{N},\beta_{R}\rangle }=\sum_{i=1}^{k}\sum_{j=1}^{m}Min(Dist(G_{i}^{miss}$, *G*_*j*_)), where $G_{i}^{miss}\in S_{miss}^{\beta_{N},\beta_{R}}$=$\beta_{R}$ − $\beta_{N}$.

**Figure 3:**
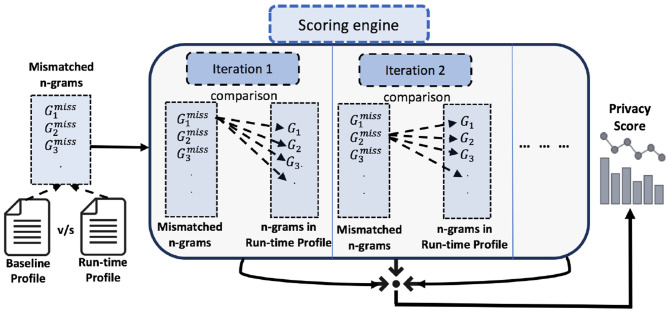
The run-time profile is compared with the baseline profile, resulting in a privacy score. Each n-gram from the set of mismatched n-grams is compared with each n-gram in the baseline profile. The minimum value of the Jaccard distance is taken from one iteration of comparison, and, subsequently, all the minimum values are added together to get the privacy score.

It is worth mentioning that the architecture of PriDe can be considered modular in nature. Therefore, one can plug in any other similarity measures for query abstractions. Example 1 shows the comparison of two n-grams. The process of profile comparison is also depicted in Figure 3.

**Example 1.** Consider the following two n-grams 〈*A*(*Q*_1_), *A*(*Q*_2_)〉 and 〈*A*(*Q*_3_), *A*(*Q*_4_)〉, where *Q*_1_, *Q*_2_, *Q*_3_, and *Q*_4_ are shown in Figure 2. The comparison of the two n-grams is as follows,

$$\begin{array}{r} Dist\left(\langle A\left(Q_{1}\right),A\left(Q_{2}\right)\rangle ,\langle A\left(Q_{3}\right),A\left(Q_{4}\right)\rangle \right)=\\ JaccardD\left(A\left(Q_{1}\right),A\left(Q_{3}\right)\right)+JaccardD\left(A\left(Q_{2}\right),A\left(Q_{4}\right)\right)\\ =\left(7-4\right)/7+\left(6-4\right)/6\\ =0.76 \end{array}$$

#### 3.2.2. Privacy Equivalence Between Attributes

Suppose the table shown in Figure 1 is queried using the SQL queries *Q*_1_, *Q*_2_, and *Q*_3_ shown in Figure 2. The records returned by *Q*_2_ are a subset of those returned by *Q*_1_, and the records returned by *Q*_2_ are also a subset of those returned by *Q*_3_ in terms of privacy. *Q*_1_ and *Q*_2_ have privacy equivalence; however, *Q*_2_ and *Q*_3_ are not privacy equivalent. The determination of privacy equivalence relations stems from the Discrimination Rate privacy metric (Sondeck et al., 2017). To define privacy equivalence relation, we first briefly review the Discrimination Rate privacy metric before furthering discussion on Privacy equivalence relation.

**Rate (DR) Privacy Metric:** the Discrimination Rate (DR) is a recently proposed privacy metric for measuring the efficiency of an anonymity system based on information theory. The Discrimination Rate privacy metric considers the attribute as a discrete random variable, while the result set is considered as the set of outcomes of another discrete random variable. For instance, consider two discrete random variables, *X* and *Y*, where *X* is the set of outcomes and *Y* is the attribute for which the measurement of the identification capacity is desired. *H*(*X*) (entropy) represents the amount of information carried by *X*. The entropy of *X* conditioned on *Y*, i.e., (*H*(*X*|*Y*)) (Sondeck et al., 2017), is computed as the measure of the effect of *Y* on *X*. Therefore, the amount of information carried by *Y* (attribute) according to *X* is given by *H*(*X*)−*H*(*X*|*Y*). Moreover, *H*(*X*)−*H*(*X*|*Y*) is divided by *H*(*X*) to get a normalized value. The Discrimination Rate of an attribute is a value in the interval [0, 1]. The Discrimination Rate value 0 for an attribute means that the attribute does not contribute to refining the attacker's knowledge when carrying out a re-identification attack.

**Definition 3.5. Discrimination Rate** Consider two discrete random variables, *X* and *Y*, where *X* is the set of outcomes and *Y* is the attribute for which the measurement of the identification capacity is desired. *H*(*X*) and (*H*(*X*|*Y*)) are the entropy of *X* and the entropy of *X* conditioned on *Y*, respectively. The discrimination rate is computed as $DR_{X}\left(Y\right)=1-\frac{H\left(X|Y\right)}{H\left(X\right)}$.

*H*(*X*) is the entropy of a discrete random variable *X* and is computed by Equation (2). *H*(*X*|*Y*) is the conditional entropy of a discrete random variable *X* given a discrete random variable *Y* and is computed by Equation (3). The discrete random variable can take values from S with probability *p*(*x*).

(2) $$\begin{array}{l} H\left(X\right)=-\underset{x\in S}{\sum }p\left(x\right)log\left(p\left(x\right)\right) \end{array}$$

(3) $$\begin{array}{l} H\left(X|Y\right)=-\underset{x\in S_{1}}{\sum }\underset{y\in S_{2}}{\sum }p\left(x,y\right)log\left(p\left(x|y\right)\right) \end{array}$$

where *p*(*x, y*)*log*(*p*(*x*|*y*) are the joint and conditional probability distributions for discrete random variables *X* and *Y*. The discrimination rate is computed for the combination of attributes and is known as the Combined Discrimination Rate (CDR). The discrimination rate value 1 for an attribute means that the knowledge of the values of this attribute leads to a re-identification attack.

**Definition 3.6. Combined Discrimination Rate (CDR)** Consider discrete random variables *X* and *Y*_1_, *Y*_2_, …*Y*_*n*_, where *X* is the set of outcomes and *Y*_1_, *Y*_2_, …*Y*_*n*_ is the set of attributes for which the measurement of the identification capacity is desired. *H*(*X*) and *H*(*X*|*Y*_1_, *Y*_2_, …*Y*_*n*_) are the entropy of *X* and the entropy of *X* conditioned on *Y*_1_, *Y*_2_, …*Y*_*n*_, respectively. The combined discrimination rate (CDR) for *Y*_1_, *Y*_2_, …*Y*_*n*_ given *X* is computed as $CDR_{X}\left(Y_{1},Y_{2},\ldots Y_{n}\right)=1-\frac{H\left(X|Y_{1},Y_{2},\ldots Y_{n}\right)}{H\left(X\right)}.$

**Figure 4:**
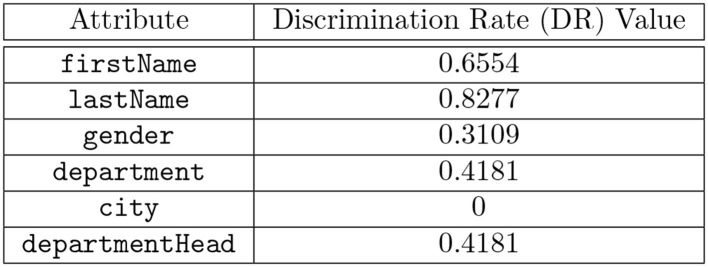
Computed Discrimination Rate (DR) values for each attribute in the table shown in Figure 1.

**Figure 5:**
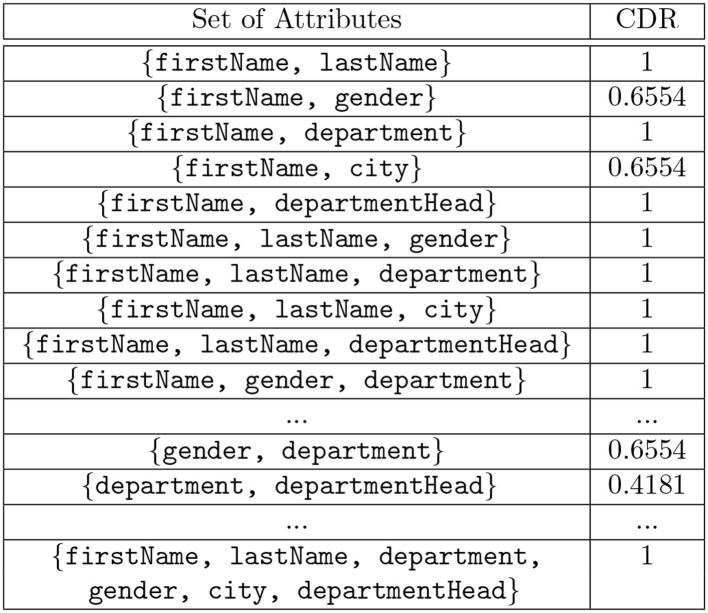
Computed Combined Discrimination Rate (CDR) values for the attributes in the table shown in Figure 1.

The discrimination rate of each attribute in the table shown in Figure 1 is shown in Figure 4 and the combined discrimination rate (CDR) for combination of attributes are shown in Figure 5. In other words, the DR (or CDR) for attributes (or a combination of attributes) is the identification capability of the attributes (or combination of attributes).

#### 3.2.3. Defining Privacy Equivalence Relation

To perform a privacy comparison between two sets of attributes, we describe a *privacy-equivalence* relation as defined in (Khan et al., 2019b). We denote the privacy equivalence relation as $\overset{p}{\equiv }$, and we generalize privacy equivalence relation for a set of attributes.

**Definition 3.7. Privacy Equivalence Relation** $\overset{p}{\equiv }$ Given two sets of attributes *S*_*i*_ and *S*_*j*_, then the privacy equivalence between *S*_*i*_ and *S*_*j*_ is defined as *S*_*i*_, being a subset of *S*_*j*_, and, for every *x* ∈ *z*, has a discrimination rate value of 0, where *z* = *S*_*j*_ ∩ *S*_*i*_ or an attribute or a set of attributes ∈ *S*_*j*_ ∪ *S*_*i*_ has *DR*_*X*_(*atr*) or *CDR*_*X*_(*atr*_1_, …, *atr*_*k*_) = 1.

A query abstraction *A*(*Q*_*i*_) is a set of elements. However, to compare the attributes in the *A*(*Q*_*i*_), we exclude the SQL command type from *A*(*Q*_*i*_) such that *S*_*Q*_*i*__ = (*A*(*Q*_*i*_) - SQL Command Type). For instance, let *A*(*Q*_*i*_) = { SELECT, lastName, gender, hospitaldb }, then *S*_*Q*_*i*__ = { lastName, gender, hospitaldb }. For the table shown in Figure 1, the Discrimination Rate for the attribute city is 0 therefore we deduce that, for the queries shown in Figure 2, *A*(*Q*_1_) and *A*(*Q*_2_) hold privacy equivalence between them, i.e., *A*(*Q*_1_) $\overset{p}{\equiv }$ *A*(*Q*_2_) while *A*(*Q*_1_) and *A*(*Q*_3_) do not hold privacy equivalence between them, i.e., *A*(*Q*_1_) $\overset{p}{≢}$ *A*(*Q*_3_) because the Discrimination Rate value for the attribute department is not zero, although the result set of *A*(*Q*_2_) is a subset of the result set of *A*(*Q*_3_). An example of the comparison of n-grams with privacy equivalence relation is shown in Example 2.

**Example 2.** Consider the following two n-grams, 〈*A*(*Q*_1_), *A*(*Q*_3_)〉 and 〈*A*(*Q*_2_), *A*(*Q*_4_)〉, where *Q*_1_, *Q*_2_, *Q*_3_, and *Q*_4_ are shown in Figure 2. We know that *A*(*Q*_2_) $\overset{p}{\equiv }$ *A*(*Q*_1_). The distance between the two above-mentioned n-grams is given by *Dist*(〈*A*(*Q*_1_), *A*(*Q*_3_)〉, 〈*A*(*Q*_2_), *A*(*Q*_4_)〉),

$$\begin{array}{r} Dist\left(\langle A\left(Q_{1}\right),A\left(Q_{3}\right)\rangle ,\langle A\left(Q_{2}\right),A\left(Q_{4}\right)\rangle \right)=\\ JaccardD\left(A\left(Q_{1}\right),A\left(Q_{2}\right)\right)+JaccardD\left(A\left(Q_{3}\right),A\left(Q_{4}\right)\right)\\ =0+\left(6-4\right)/6\\ =0.33 \end{array}$$

     □

#### 3.2.4. The Scenario of Cold Start

It is worthwhile to consider the scenario of a *cold start*—the unavailability of a baseline profile. In this scenario, only a run-time profile is generated, and the privacy score is computed solely using this run-time profile. One can think of the comparison in the cold start scenario as comparing the run-time profile with an empty (baseline) profile. The privacy score in this scenario is the “*total number of unique n-grams* × *n*,” where *n* is the size of the n-gram in the run-time profile, i.e., |$\beta_{R}$| × *n*. However, this is not the case when we look into the cumulative privacy score, which is discussed in section 3.3.

### 3.3. Cumulative Privacy Score

It could be the case that the user of the system wants to add the privacy scores of several days in order to get an idea of how much loss of privacy there was in those days in total. The privacy scores cannot be added to each other in naïve way. This section describes how the privacy scores can be summed.

To compute the privacy score, one generates a baseline profile and a run-time profile and, subsequently, compares the profiles with each other. The question that arises is, “when should the run-time profile be constructed?” As for the construction of the baseline profile, one can construct it before the information system is up and running. On the other hand, the run-time profile is constructed when the information system is operational, meaning that access has been granted to the analyst, and the analyst has started making queries. The time horizon can be divided into equal intervals so that run-time profiles can be constructed for each interval. The time horizon is the total time period for which the analyst was granted access to the database. For simplicity, we opt to construct a run-time profile by the end of each day. That is to say, audit logs are collected by the end of each day, and then the run-time profile is generated using these logs. We denote the run-time profile constructed by the end of the *x*th day as β_*X*_; for example, the run-time profile constructed by the end of day 1 is denoted as β_1_. β_0_ represents the profile constructed on day 0 or the baseline profile. It is worth mentioning that the baseline profile is generated only once. We denote the privacy score computed on the *x*^*th*^ day as *P*_〈_β_0__, β_*X*_〉_; for example, the score computed on day 2 is denoted as *P*_〈β_0_, β_2_〉_. In the cold start scenario, where the baseline profile is unavailable, we denote the privacy score as *P*_〈{},β_1_〉_ computed on day 1 and, similarly, the privacy score computed on day 2 as *P*_〈{},β_2_〉_, and so forth.

Imagine that the owner of the information gets a privacy score for each day but desires the cumulative privacy score at day 10. A naïve addition of privacy scores for all 10 days (from day 1 to 10) is not an accurate representation of the cumulative privacy score. For instance, a user can make identical queries in the same order every day for 10 days, thus resulting in an identical n-gram profile for each day. Therefore, one must take into account the combination of n-gram profiles for 10 days in such a way that only unique behaviors (unique n-grams) for all 10 days are part of the combined n-gram profile of all 10 days. To unravel this, we define the cumulative privacy score, denoted as Δ.

**Definition 3.8. Cumulative Privacy Score** Let the cumulative privacy score from day 1 to day *X* be denoted as Δ[< β_0_ >, < β_1_, β_2_, …, β_*X*_ >], where < β_1_, β_2_, …, β_*X*_ > = (β_1_ ∪ β_2_ ∪ β_3_ ∪ β_4_, …, ∪ β_*X*_)=β_(1, 2, …, *X*)_. The cumulative privacy score is computed as follows: Δ[< β_0_ >, < β_1_, β_2_, …., β_*X*_ >] = $\sum_{i=1}^{k}\sum_{j=1}^{m}Min(Dist(G_{i}^{miss}$, *G*_*j*_)) where $G_{i}^{miss}\in S_{miss}^{\beta_{0},\beta_{\left(1,2,\ldots ,X\right)}}$ = β_(1, 2, …, *X*)_−β_0_.

**Figure 6:**
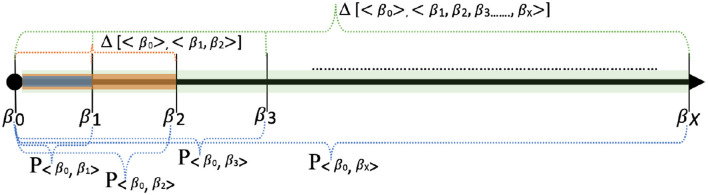
Variations of privacy score computations: This figure shows a variety of ways in which individual privacy scores and cumulative privacy scores can be computed. For example, Δ[< β_0_ >, < β_1_, β_2_ >] represents the cumulative privacy for day 1 and day 2. *P*_〈β_0_,β_3_〉_ represents the individual privacy score for day 3.

The union defined over the profiles considers the profiles as sets, with n-grams being the elements of the sets. The algorithm for privacy score computation where the baseline profile is available is shown in Algorithm 1. Figure 6 shows some scenarios of the privacy score computation with respect to a baseline profile (reference point). In the case of the cold start scenario, the cumulative privacy score for day 1 to day 3 is denoted as Δ[< {} >, < β_1_, β_2_, β_3_ >]. The cumulative privacy score in the cold start scenario from day 1 to day *X* is computed as follows: |β_1_ ∪ β_2_ ∪, …, β_*X*_| × size of n-gram.

**Algorithm 1:**
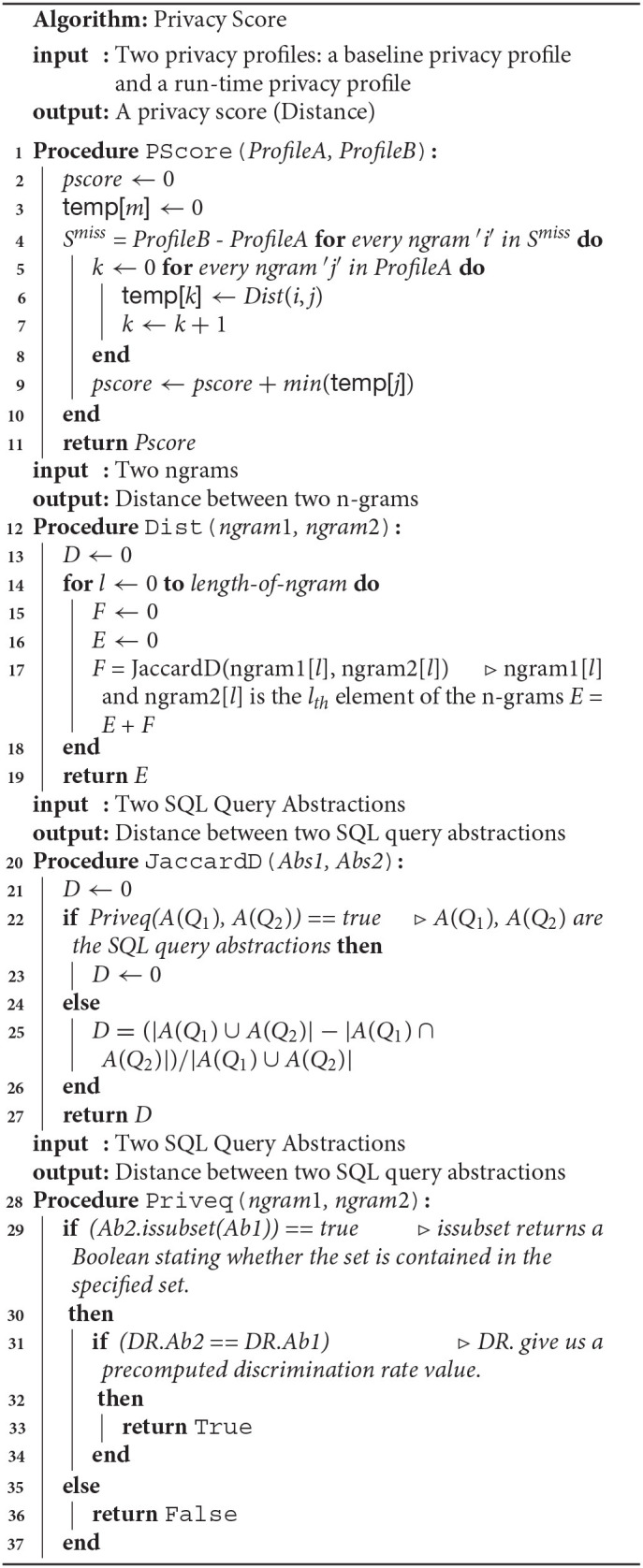
The privacy score algorithm, *Pscore*, takes two privacy profiles as an input and returns a privacy score. The algorithm *Dist* computes the similarity between the two n-grams.

#### 3.3.1. Max (Worst-Case) Privacy Score

One (as the data curator) could be interested in knowing what the maximum or worst-case privacy score possible would be. The availability of a worst-case privacy score enables the data curator to make well-informed decisions by comparing the worst-case privacy score with the actual privacy score. For instance, an insignificant difference between the worst-case privacy score and the actual privacy score is indicative of potentially malicious querying behavior. The Max (worst-case) privacy score is denoted by ${\text{M}}_{\beta_{X}}$. In the proposed model, the maximum privacy score (worst-case privacy score) is the *total number of unique n-grams* × *n*, where *n* is the size of the n-gram in the run-time profile, i.e., ${\text{M}}_{\beta_{X}}=|\beta_{R}|\times n$.

#### 3.3.2. Acceptable Threshold for Privacy-Loss Score

A challenge in the privacy-score model, and in general, is how to determine what is an acceptable privacy-loss score^2^. There are two ways of finding a threshold. The first way to compare it is with reference to the worst-case privacy-loss score. The second way is to compare it with past privacy-loss scores. In future, we are interested in examining how to determine an acceptable threshold for a privacy-loss score, especially in the scenario of cold start.

## 4. Computing Privacy Score

We evaluated the proposed approach in two scenarios. One scenario was of an application where generic users (or roles of users) have a standard behavior. As a result, a baseline profile can be constructed. The other contrasting scenario is a cold start. For the first scenario, we considered a synthetic banking application for managing accounts, that is, a transaction-oriented system. Reasonably, a user with a role in the bank has similar behavior to other users with the same role. For the cold start scenario, we considered health-care predictive analytics settings where users look up information in the hospital database to gain insights. In the cold start scenario, when a new user is appointed, a baseline profile is unavailable for their behavior. The reason for using synthetic applications is because it is hard to gain access to real-world data-sets. Organizations are hesitant to and are sometimes legally constrained from sharing sensitive data (Sallam and Bertino, 2017), especially in the era of GDPR. Benchmark data-sets (query logs) consisting of a variety of SQL queries made to a DBMS for diverse scenarios are highly desirable to facilitate research in this domain. Admittedly, the unavailability of real-world data-sets is a significant constraint in the evaluation of the approaches proposed in this line of research and is vexing to researchers working in it.

**Figure 7:**
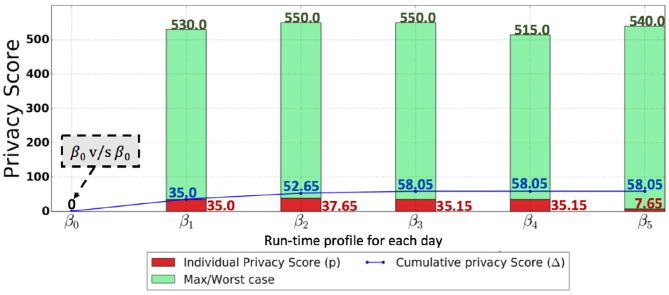
This graph shows the results for the scenario of a banking setting, with the SQL query abstraction in Figure 2 being used. The red bar in the figure shows the actual privacy score, while the green bar in the figure indicates the maximum possible privacy score (worst-case privacy score—${\text{M}}_{\beta_{X}}$) for each day. The blue line shows the cumulative privacy score.

**Figure 8:**
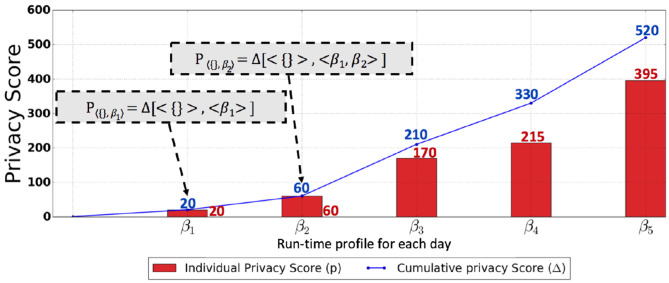
This graph shows the results of the cold start scenario using the SQL query abstraction depicted in Figure 2. The red bar shows the actual privacy score, and the blue line shows the cumulative privacy score over the time horizon.

The audit logs of the banking application consisted of transactions that include account open, account close, withdraw, deposit, and transfer. Each transaction is initiated by an employee of the bank with a role and involves the execution of a number of SQL statements. Audit logs for 6 days, i.e., the audit logs of day 0, day 1, day 2, …, day 5, were collected. The audit log of day 0 was used to generate a baseline profile. The application system was run with 2500 random transactions for each day, in total generating around 7200 SQL statements for each audit log. In the health-care predictive analytics setting, audit logs for day 1, day 2, …, day 5 were collected, as day 0 was undesired in the cold start scenario. Each audit log consisted of computer-generated queries such that more attributes (combination of attributes) were queried as compared to the attributes (combination of attributes) queried the previous day. The audit log of day 1, day 2, day 3, day 4, and day 5 consisted of around 700, 1200, 2200, 3000, and 5500 queries. We denote the profiles constructed from the audit log of each day as β_*day*_. The profiles constructed on day 0, day 1, day 2, day 3, day 4, and day 5 were denoted as β_0_, β_1_, β_2_, β_3_, β_4_, and β_5_ for both scenarios. PriDe was deployed in both the scenarios, and both the individual privacy score and the cumulative score are shown for both scenarios in Figure 7 (with baseline profile) and Figure 8 (cold start scenario).

Figure 7 shows the scenario of the banking setting, where the SQL query abstraction in Figure 2 is used. The individual privacy scores in a banking setting for day 1, day 2, day 3, day 4, and day 5 are 35.0, 37.65, 35.15, 35.15, and 7.65, respectively. The red bar in Figure 7 shows the actual privacy score, while the green bar in Figure 7 indicates the max (worst-case) privacy score ${\text{M}}_{\beta_{X}}$. These privacy scores for each day indicate that there are newly discovered querying behaviors resulting in a reduction of privacy with respect to the attribute values previously retrieved by the user. However, the privacy score for each day when compared to the potential worst-case privacy score is insignificant, particularly for day 5. On the other hand, from the cumulative privacy score, one can obtain more meaningful insights. The cumulative score for day 1 indicates the discovery of new querying behavior resulting in a reduction of privacy; nevertheless, a further reduction in privacy is indicated by a minor increase in cumulative privacy score for day 2. The small increase in cumulative privacy score on day 3 implies that there are further new behaviors discovered apart from the ones discovered on days 1 and 2, thus indicating a further reduction in privacy. Identical cumulative privacy scores for days 3, 4, and 5 indicate that, at this point, the unknown querying behaviors have already been discovered; however, these querying behaviors are repeated on days 3, 4, and 5, as indicated by the individual privacy scores. This kind of trend was expected because of the nature of the banking application, where a set of identical transactions are repeated daily, resulting in less diverse SQL queries and thus an insignificant individual privacy score each day and a stable cumulative privacy score over several days, provided that the baseline profile is well captured. However, an unexpected increase in cumulative privacy score, as well as in individual privacy score, is an indication of peculiarities. Figure 8 shows the individual and cumulative privacy scores in a health-care predictive analytics setting. The individual privacy scores for day 1, day 2, day 3, day 4, and day 5 are 20, 60, 170, 215, and 395, respectively. In contrast with the banking setting, the baseline profile is not present; additionally, the audit logs do not consist of repeated transactions. In contrast to the cumulative privacy score in the banking setting, the cumulative privacy score here is significantly increased each day. The cumulative privacy scores on day 1 and day 2 are the same as the individual privacy scores, indicating that querying behavior on day 1 was repeated on day 2 along with new querying behaviors resulting in the reduction of privacy. There is an increasing trend in cumulative privacy score for day 3, day 4, and day 5; these cumulative privacy scores are higher than the corresponding day's individual privacy score, indicating a reduction of privacy with respect to the attribute values retrieved on day 1 and day 2 by the analyst from the database. The increasing trend in cumulative privacy score is an indication that the user is making diverse queries, and this is being validated by the cumulative privacy score.

### 4.1. Use-Case: Global Consistency

The privacy-loss score is more suited in the cold start scenario. However, there are scenarios where the distance between the two behaviors is relevant for calculating the privacy loss, for instance, for global consistency. The proposed approach for computing a quantitative score for privacy can serve as a good tool to provide a global consistency measure. In case where several data analytics firms are simultaneously granted access to the organization's database, the organization (data curator) can perform a consistency check using a privacy score to monitor and subsequently can take measures before a breach materializes.

An enterprise, Ozon (the data curator), collected a huge amount of data over some period of time. The data consists of the purchases made via its online shopping portal. Motivated by the financial gain, Ozon decided to grant access to its application data to many companies, namely Acme Analytics, Wayne Analytics, and Start Analytics. Following the defense-in-depth strategy, Ozon already had a number of security controls in place. Due to growing concerns over data privacy, this time, Ozon's management reached a decision to increase a layer by adding a technology, PriDe, that monitors information gain in a privacy sense. Granting access to third parties has been a regular practice by Ozon, and no privacy incidents have taken place in the past. However, Ozon has a policy of keeping the audit logs of all interactive sessions when access to its data is granted. Using these past logs with PriDe, Ozon integrated PriDe into its privacy and security dashboards. When the third parties performed their analytics, Ozon kept an eye on the privacy scores of these third parties so as to have a consistency check to monitor for peculiarities; for instance, if *Acme Analytics*'s privacy score is much higher than that of the rest of the firms, then this alerts Ozon to take urgent measures before any unforeseen breach materializes.

### 4.2. Modeling Querying Behavior Using Machine Learning

The proposed privacy-loss score approach uses n-grams to capture short-term correlations between the queries made by the user to the DBMS. In essence, the n-grams are being used to model the querying behavior of the user. However, this is only one of the ways in which querying behavior can be modeled. Another way to model a user's querying behavior is to use machine learning. Machine learning has been applied in behavioral analysis for the detection of misbehavior in VANET (Grover et al., 2011), modeling driver's behavior using smartphone sensor data (Ferreira et al., 2017), and modeling behavior models for Android Malware Analysis (Chuang and Wang, 2015). A number of machine learning-based approaches exist for modeling behaviors within specific applications; however, little attention has been paid to machine learning-based approaches in the context of modeling query behavior in terms of privacy, thus enabling one to compute a privacy-loss score. As future work, we are interested in exploring the interpretation of machine learning for behavioral analysis to model querying behaviors in terms of privacy.

### 4.3. Application in Detection of SQL Injections

Detection of malicious queries, including SQL injections, is a significant area of research (McWhirter et al., 2018). The privacy-loss score model is built upon another model that captures the querying behavior of a user and constructs user profiles. Given a normative profile of a user constructed using safe queries (where, by safe, we mean queries free from any malicious queries including SQL injections), we compare this with a run-time profile that may have malicious queries including SQL injections and label potentially malicious queries and SQL injections as mismatches (anomalies). For the sake of clarity, we refer to this application of a part of the privacy-loss score model as the Malicious Query Detection (MalDetect) approach. The proposed approach in McWhirter et al. (2018) uses a machine learning-based solution of classifying SQL queries using features of the initial query string and predicts a class for an incoming query, that is, whether the incoming query is malicious or is a normal query. Both the approach in McWhirter et al. (2018) and MalDetect, though different in construction, have the potential to achieve a high detection rate.

## 5. Conclusions

In this work, we introduced PriDe, which computes a privacy score within the framework of a Relational Database Management System (RDBMS). A strategy for a refined comparison (measuring privacy distance) in terms of privacy equivalence has also been proposed in this paper. The privacy score is computed using audit logs containing SQL queries. This makes the proposed approach stand out as, first, it quantifies the privacy risk and, second, it only requires SQL statements to compute the privacy score. Experiments were carried out to evaluate the approach in two scenarios where, in one scenario, a data-set was available for constructing the baseline profile while, in the second scenario, there was a cold start. The results suggest that PriDe can provide a quantitative score in terms of privacy that enables organizations to monitor and gain insights about the data that is being shared with a third party from a privacy perspective. The privacy score can augment a privacy dashboard that indicates the health of the system in terms of privacy, that is to say, it enables a data curator to check whether something is wrong with the way the database is being accessed. The proposed approach, without loss of generality, is applicable to BigSQL-style technologies. In future work, we would like to examine existing machine learning techniques for behavioral analysis so that they can be applied to model querying behaviors in terms of privacy. In addition to that, we would have liked to explore the translation of the privacy score model, beyond relational models, onto other data models.

## Data Availability Statement

The raw data supporting the conclusions of this article will be made available by the authors, without undue reservation, to any qualified researcher.

## Author Contributions

All authors listed have made a substantial, direct and intellectual contribution to the work, and approved it for publication.

## Conflict of Interest

The authors declare that the research was conducted in the absence of any commercial or financial relationships that could be construed as a potential conflict of interest.

## Footnotes

^1^The terms “analyst,” “principal,” and “user” are used interchangeably in this paper.

^2^The privacy score is manifestation of loss of privacy, therefore, the terms privacy score and privacy-loss score are used interchangeably.

## References

[B1] Aligon J. Golfarelli M. Marcel P. Rizzi S. Turricchia E. (2014). Similarity measures for olap sessions. Knowl. Inform. Syst. 39, 463–489. 10.1007/s10115-013-0614-1

[B2] Alizadeh M. Peters S. Etalle S. Zannone N. (2018). Behavior analysis in the medical sector: theory and practice, in Proceedings of the 33rd Annual ACM Symposium on Applied Computing, SAC '18, (New York, NY: ACM), 1637–1646. 10.1145/3167132.3167307

[B3] Arnau J. P. Rebollo-Monedero D. Forne J. (2014). Measuring the privacy of user profiles in personalized information systems. Fut. Gener. Comput. Syst. 33, 53–63. 10.1016/j.future.2013.01.001

[B4] Becker J. Chen H. (2009). Measuring privacy risk in online social networks, in Proceedings of the Workshop on Web 2.0 Security and Privacy (Oakland, CA).

[B5] CERT (2014). 2014 US State of Cybercrime Survey. Technical report, Software Engineering Institute; Carnegie Mellon University.

[B6] Chandola V. Banerjee A. Kumar V. (2009). Anomaly detection: a survey. ACM Comput. Surv. 41, 15, 1–15:58. 10.1145/1541880.1541882

[B7] Chuang H. Wang S. (2015). Machine learning based hybrid behavior models for android malware analysis, in 2015 IEEE International Conference on Software Quality, Reliability and Security (Vancouver, BC), 201–206. 10.1109/QRS.2015.37

[B8] Clifton C. Tassa T. (2013). On syntactic anonymity and differential privacy, in 2013 IEEE 29th International Conference on Data Engineering Workshops (ICDEW) (Brisbane, QLD), 88–93. 10.1109/ICDEW.2013.6547433

[B9] Damashek M. (1995). Gauging similarity with n-grams: language-independent categorization of text. Science 267, 843–848. 10.1126/science.267.5199.843 17813910

[B10] Deng Z.-H. Lv S.-L. (2015). Prepost+: An efficient n-lists-based algorithm for mining frequent itemsets via children-parent equivalence pruning. Expert Syst. Appl. 42, 5424–5432. 10.1016/j.eswa.2015.03.004

[B11] Diaz C. (2006). Anonymity metrics revisited, in Anonymous Communication and its Applications, number 05411 in Dagstuhl Seminar Proceedings, eds Dolev S. Ostrovsky R. Pfitzmann A. (Dagstuhl: Internationales Begegnungs- und Forschungszentrum für Informatik (IBFI); Schloss Dagstuhl).

[B12] Díaz C. Seys S. Claessens J. Preneel B. (2003). Towards measuring anonymity, in Privacy Enhancing Technologies, eds Dingledine R. Syverson P. (Berlin; Heidelberg: Springer Berlin Heidelberg), 54–68.

[B13] Dwork C. (2008). Differential privacy: a survey of results, in Theory and Applications of Models of Computation, eds Agrawal M. Du D. Duan Z. Li A. (Berlin; Heidelberg: Springer Berlin Heidelberg), 1–19. 10.1007/978-3-540-79228-4_1

[B14] Ferreira J. Jr. Carvalho E. Ferreira B. V. de Souza C. Suhara Y. Pentland A.. (2017). Driver behavior profiling: an investigation with different smartphone sensors and machine learning. PLoS ONE 12:e174959. 10.1371/journal.pone.0174959 28394925 PMC5386255

[B15] Forrest S. Hofmeyr S. A. Somayaji A. Longstaff T. A. (1996). A sense of self for unix processes, in Proceedings 1996 IEEE Symposium on Security and Privacy (Oakland, CA), 120–128.

[B16] Genga L. Zannone N. (2018). Towards a systematic process-aware behavioral analysis for security, in Proceedings of the 15th International Joint Conference on e-Business and Telecommunications, ICETE 2018 - Volume 1: DCNET, ICE-B, OPTICS, SIGMAP and WINSYS (Porto), 626–635.

[B17] Grover J. Prajapati N. K. Laxmi V. Gaur M. S. (2011). Machine learning approach for multiple misbehavior detection in vanet, in Advances in Computing and Communications, eds Abraham A. Mauri J. L. Buford J. F. Suzuki J. Thampi S. M. (Berlin; Heidelberg: Springer Berlin Heidelberg), 644–653.

[B18] Halkidi M. Koutsopoulos I. (2011). A game theoretic framework for data privacy preservation in recommender systems, in Machine Learning and Knowledge Discovery in Databases, D. Gunopulos, T. Hofmann, D. Malerba, and M. Vazirgiannis (Berlin; Heidelberg: Springer Berlin Heidelberg), 629–644.

[B19] Hofmeyr S. A. Forrest S. Somayaji A. (1998). Intrusion detection using sequences of system calls. J. Comput. Secur. 6, 151–180. 10.3233/JCS-980109

[B20] Hussain S. R. Sallam A. M. Bertino E. (2015). Detanom: detecting anomalous database transactions by insiders, in Proceedings of the 5th ACM Conference on Data and Application Security and Privacy, CODASPY '15 (New York, NY: ACM), 25–35.

[B21] Kalchbrenner N. Grefenstette E. Blunsom P. (2014). A convolutional neural network for modelling sentences, in Proceedings of the 52nd Annual Meeting of the Association for Computational Linguistics (Baltimore, MD).

[B22] Khan M. I. Foley S. N. (2016). Detecting anomalous behavior in dbms logs, in In International ference on Risks and Security of Internet and Systems (CRiSIS2016) (Roscoff), 147–152.

[B23] Khan M. I. Foley S. N. O'Sullivan B. (2019c). PriDe: a quantitative measure of privacy-loss in interactive querying settings, in 2019 10th IFIP International Conference on New Technologies, Mobility and Security (NTMS) (Canary Islands: IEEE). 10.1109/NTMS.2019.8763781

[B24] Khan M. I. O'Sullivan B. Foley S. N. (2019a). Dbms log analytics for detecting insider threats in contemporary organizations, in Security Frameworks in Contemporary Electronic Government, eds Abassi R. Douss A. B. C. (IGI Global), ch. 10, 207–234.

[B25] Khan M. I. O'Sullivan B. Foley S. N. (2019b). Computing the identification capability of sql queries for privacy comparison, in Proceedings of the ACM International Workshop on Security and Privacy Analytics, ser. IWSPA '19. (New York, NY: ACM), 47–52.

[B26] Koh Y. S. Rountree N. (2005). Finding sporadic rules using apriori-inverse, in Advances in Knowledge Discovery and Data Mining, eds Ho T. B. Cheung D. Liu H. (Berlin; Heidelberg: Springer Berlin Heidelberg), 97–106.

[B27] Kul G. Luong D. Xie T. Coonan P. Chandola V. Kennedy O.. (2016). Ettu: Analyzing query intents in corporate databases, in Proceedings of the 25th International Conference Companion on World Wide Web, WWW '16 Companion (Republic and Canton of Geneva: International World Wide Web Conferences Steering Committee), 463–466.

[B28] Kul G. Luong D. T. A. Xie T. Chandola V. Kennedy O. Upadhyaya S. (2018). Similarity measures for SQL query clustering. IEEE Trans. Knowl. Data Eng. 10.1109/TKDE.2018.2831214

[B29] Lee S. Y. Low W. L. Wong P. Y. (2002). Learning fingerprints for a database intrusion detection system, in Proceedings of the 7th European Symposium on Research in Computer Security, ESORICS '02, (London: Springer-Verlag), 264–280.

[B30] Li N. Li T. Venkatasubramanian S. (2007). t-closeness: privacy beyond k-anonymity and l-diversity, in 2007 IEEE 23rd International Conference on Data Engineering (Istanbul), 106–115.

[B31] Low W. L. Lee J. Teoh P. (2002). Didafit: Detecting intrusions in databases through fingerprinting transactions, in ICEIS (Ciudad Real), 121–128.

[B32] Machanavajjhala A. Kifer D. Gehrke J. Venkitasubramaniam M. (2007). L-diversity: Privacy beyond k-anonymity. ACM Trans. Knowl. Discov. Data 1:52. 10.1145/1217299.1217302

[B33] Makiyama V. H. Raddick J. Santos R. D. C. (2015). Text mining applied to SQL queries: a case study for the SDSS skyserver, in Proceedings of the 2nd Annual International Symposium on Information Management and Big Data - SIMBig 2015 (Cusco), 66–72.

[B34] Mathew S. Petropoulos M. Ngo H. Q. Upadhyaya S. (2010). A data-centric approach to insider attack detection in database systems, in Proceedings of the 13th International Conference on Recent Advances in Intrusion Detection, RAID'10 (Berlin; Heidelberg: Springer-Verlag), 382–401.

[B35] McWhirter P. R. Kifayat K. Shi Q. Askwith B. (2018). SQL injection attack classification through the feature extraction of SQL query strings using a gap-weighted string subsequence kernel. J. Inform. Secur. Appl. 40, 199–216. 10.1016/j.jisa.2018.04.001

[B36] Pei J. Han J. Lu H. Nishio S. Tang S. Yang D. (2007). H-mine: fast and space-preserving frequent pattern mining in large databases. IIE Trans. 39, 593–605. 10.1080/07408170600897460

[B37] Phillips J. M. (2015). Lecture Notes: Jaccard Similarity and k-Grams. University of Utah.

[B38] Sallam A. Bertino E. (2017). Detection of temporal insider threats to relational databases, in 2017 IEEE 3rd International Conference on Collaboration and Internet Computing (CIC), 406–415.

[B39] Sallam A. Fadolalkarim D. Bertino E. Xiao Q. (2016). Data and syntax centric anomaly detection for relational databases. Wiley Interdisc. Rev. 6, 231–239. 10.1002/widm.1195

[B40] Serjantov A. Danezis G. (2003). Towards an information theoretic metric for anonymity, in Privacy Enhancing Technologies, eds Dingledine R. Syverson P. (Berlin; Heidelberg. Springer Berlin Heidelberg), 41–53.

[B41] Shapira B. Elovici Y. Meshiach A. Kuflik T. (2004). PRAW a privacy model for the web. J. Am. Soc. Inform. Sci. Technol. 56, 159–172. 10.1002/asi.20107

[B42] Sidorov G. Velasquez F. Stamatatos E. Gelbukh A. Chanona-Hernandez L. (2014). Syntactic n-grams as machine learning features for natural language processing. Expert Syst. Appl. 41, 853–860. 10.1016/j.eswa.2013.08.015

[B43] Somayaji A. Forrest S. (2000). Automated response using system-call delays, in Proceedings of the 9th Conference on USENIX Security Symposium - Volume 9, SSYM'00 (Berkeley, CA: USENIX Association), 14.

[B44] Sondeck L. P. Laurent M. Frey V. (2017). Discrimination rate: an attribute-centric metric to measure privacy. Ann. Telecommun. 72, 755–766. 10.1007/s12243-017-0581-8

[B45] Stefanidis K. Drosou M. Pitoura E. (2009). You may also like results in relational databases, in Proceedings International Workshop on Personalized Access. Profile Management and Context Awareness Databases (PersDB 2009), in conjunction with VLDB 2009 (Lyon).

[B46] Sweeney L. (2002). K-anonymity: a model for protecting privacy. Int. J. Uncertain. Fuzziness Knowl.-Based Syst. 10, 557–570. 10.1142/S0218488502001648

[B47] Uno T. Kiyomi M. Arimura H. (2004). LCM ver.2: efficient mining algorithms for frequent/closed/maximal itemsets, in Proc. 1st Int'l Workshop on Open Source Data Mining: Frequent Pattern Mining Implementations (Brighton).

[B48] Wagner I. Boiten E. A. (2017). Privacy risk assessment: from art to science, by metrics. arXiv:1709.03776 [cs.CR]. 10.1007/978-3-030-00305-0_17

[B49] Zhang N. Zhao W. (2007). Privacy-preserving data mining systems. Computer 40, 52–58. 10.1109/MC.2007.142

